# Supplementary material for: Mitochondria-Targeted Fluorescent Nanoparticles with Large Stokes Shift for Long-Term BioImaging
Source: Molecules. 2023 May 8;28(9):3962. doi: 10.3390/molecules28093962 (PMC10179964; doi:10.3390/molecules28093962)
Supplement: Supplementary file 1 [file molecules-28-03962-s001.zip › molecules-2297443-Supplementary.pdf]

molecules

MDPI

Article

Mitochondria-Targeted Fluorescent Nanoparticles with Large Stokes Shift for Long-Term BioImaging

Xiao Li [1], Tao Zhang [1], Xuebo Diao [2], Yu Li [3], Yue Su [4], Jiapei Yang [4], Zibo Shang [5], Shuai Liu [1], Jia Zhou [6], Guolin Li [1,7,*] and Huirong Chi [7,*]

Apparatus

Nuclear magnetic resonance (NMR) spectra were recorded on Bruker AVANCE III 400 spectrometer with dimethyl sulfoxide-d6 (DMSO-d_6) as the solvents at 25°C. FTIR spectra were recorded on a Paragon 1000 instrument by the KBr sample holder method. DLS measurements were recorded on a Malvern Zetasizer Nano S device with a 4.0 mW laser operating at λ=633 nm. All samples of 0.1 mg mL^{-1} were tested with a scattering angle of 173°at 37°C. TEM studies were performed with a JEM-2010HT instrument operated at 200 kV to observe the shape and size of the micelles. Samples were prepared by directly dropping the solution onto carbon-coated copper grids and then air-drying at room temperature overnight before measurement. The UV-vis spectra were measured on a Perkin-Elmer Lambda 20/2.0 UV-vis spectrophotometer. At room tempreture, the fluorescence spectra were recorded on a QM/TM/RM fluorescence spectrophotometer (Photon Technology International, Inc.). Molecular weight was determined through a MS system (Agilent).

Materials

4-methoxy-o-Phenylenediamine (98%, Adamas), ethyl pyruvate (99%, Adamas), K_2CO_3 (99.0%, Greagent), Acetone (99.5%, Adamas), 3-bromoprop-1-ene (95%, Matrix), ethyl acetate (99.8%, Adamas), $MgSO_4$ (Fluka, 99.0%), N-(4-formylphenyl)acetamide (Aladdin, 99.0%), sulfuric acid (96%, Adamas), (E)-4-bromobut-2-enoic acid (Aladdin, 99.0%), N-hydroxysuccinimide (NHS, 99%, Adamas), 3-(4,5-dimethyl-thiazol-2-yl)-2,5-diphenyl tetrazolium bromide (MTT, Sigma), anhydrous ethanol and peptide (AC-Kr-(Cha)-r-(Cha)-r-(Cha)-r)(Remark:r is D-Arg),98%, GL Biochem) were used as received without purification. DMEM culture medium, and fetal bovine serum (FBS) were purchased from Gibco.L929 mouse fibroblast and IOSE-80 human ovarian epithelium cells were purchased from ATCC

A

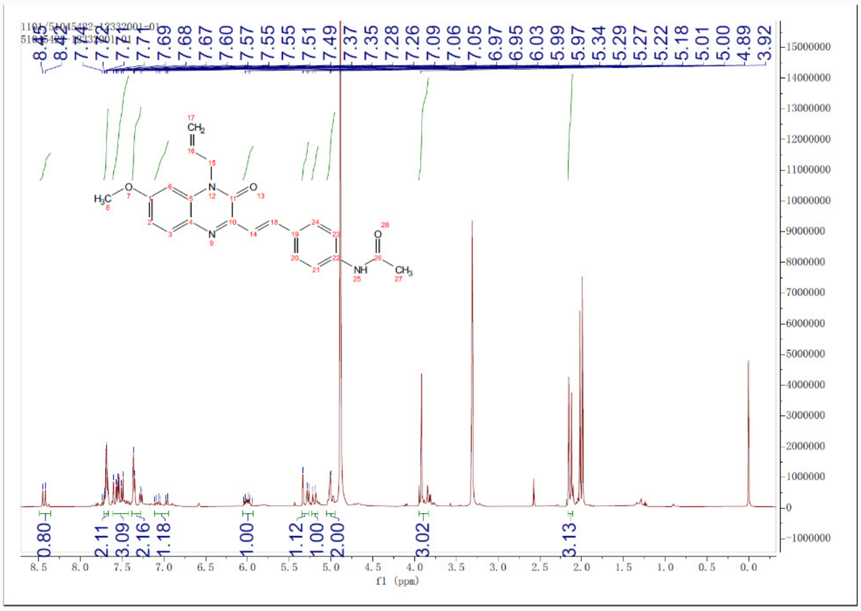
7.72 7.71 7.69 7.68 7.67 7.60 7.57 7.55 7.55 7.51 7.49 7.37 7.35 7.28 7.26 7.09 7.06 7.05 6.97 6.95 6.03 5.99 5.97 5.34 5.29 5.27 5.22 5.18 5.01 5.00 4.89 3.92
0.80 2.11 3.09 2.16 1.18 1.00 1.12 1.00 2.00 3.02 3.13
f1 (ppm)

B

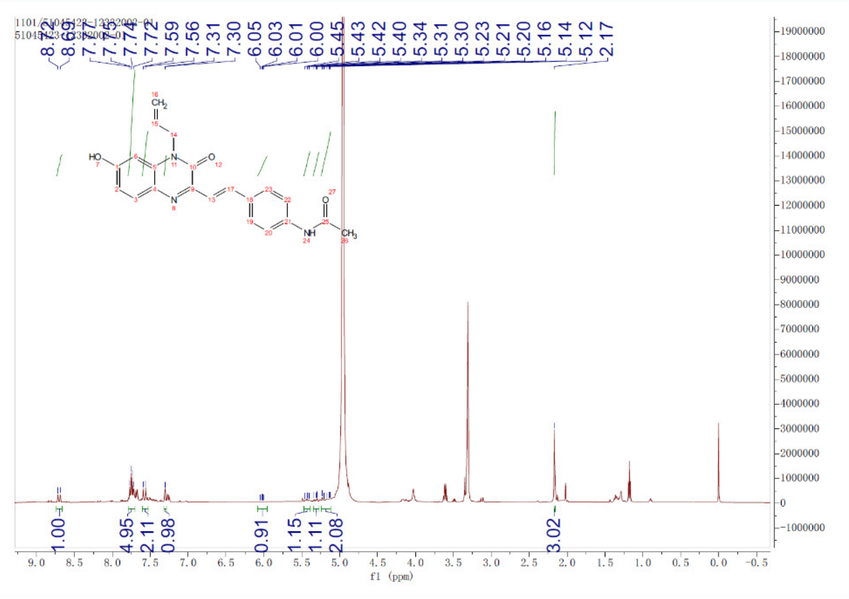
7.72 7.59 7.56 7.31 7.30 6.05 6.03 6.01 6.00 5.45 5.43 5.42 5.40 5.34 5.31 5.30 5.23 5.21 5.20 5.16 5.14 5.12 2.17
1.00 4.95 2.11 0.98 0.91 1.15 1.11 2.08 3.02
f1 (ppm)

C

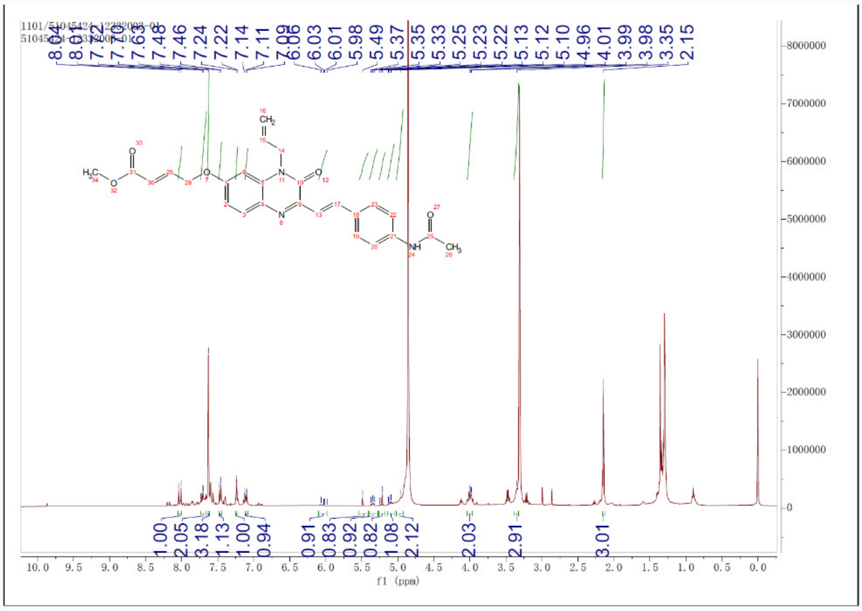
7.48 7.46 7.24 7.22 7.14 7.11 6.03 6.01 5.98 5.49 5.37 5.35 5.33 5.25 5.23 5.22 5.13 5.12 5.10 4.96 4.01 3.99 3.98 3.35 2.15
1.00 2.05 3.18 1.13 1.00 0.94 0.91 0.83 0.92 0.82 1.08 2.12 2.03 2.91 3.01
f1 (ppm)

Figure S1. ^{1}H NMR spectra of Compound 7(A), Compound 8(B) and Compound 9(C) .

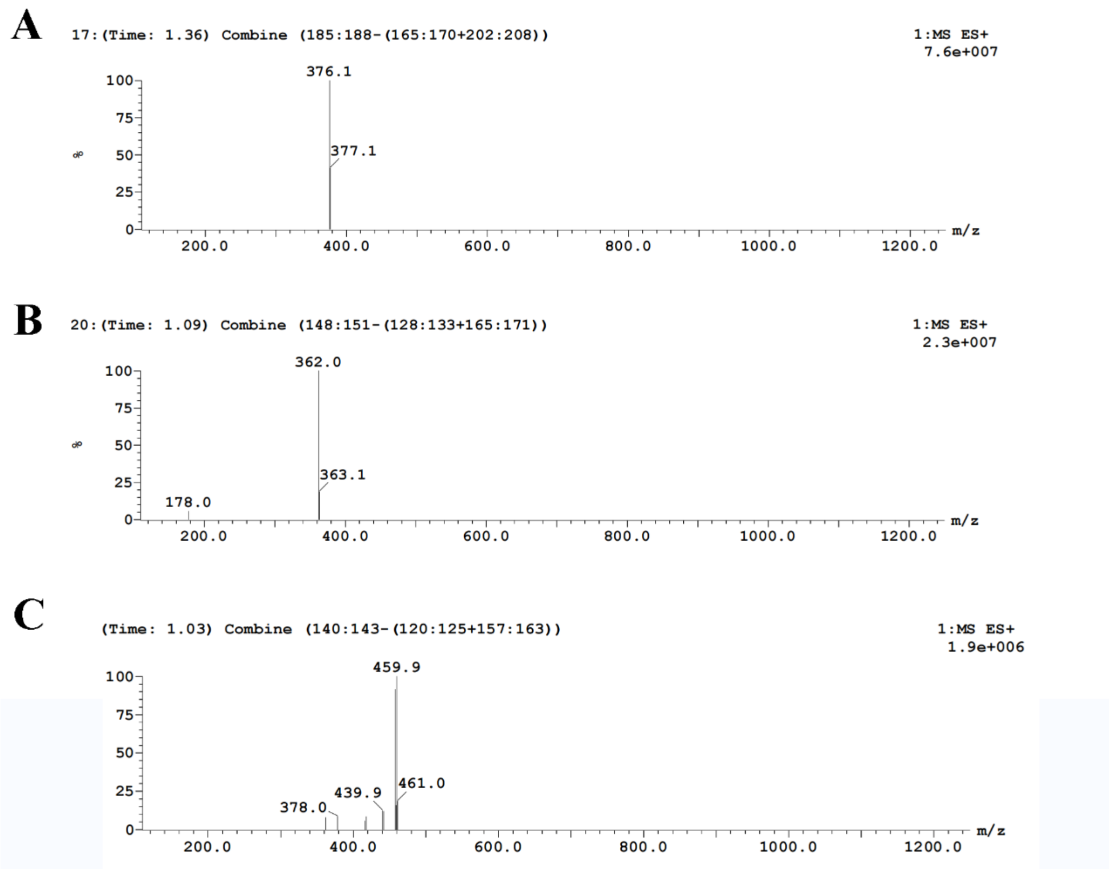

Figure S2. MS spectra of Compound 7(A), Compound 8(B) and Compound 9(C) .

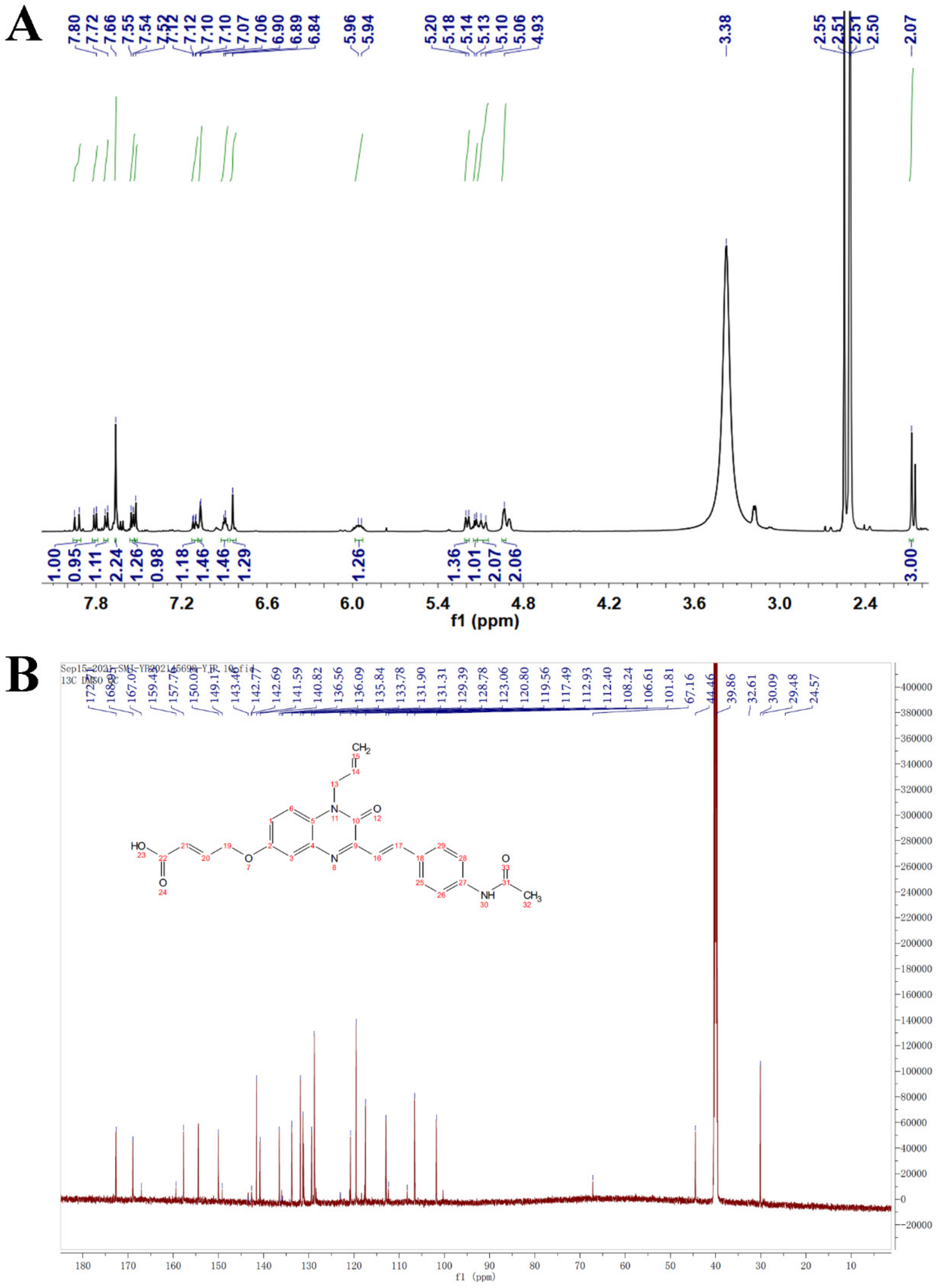

Figure S3. ^{1}H NMR spectra of QC (A). ^{13}C NMR spectra of QC (B).

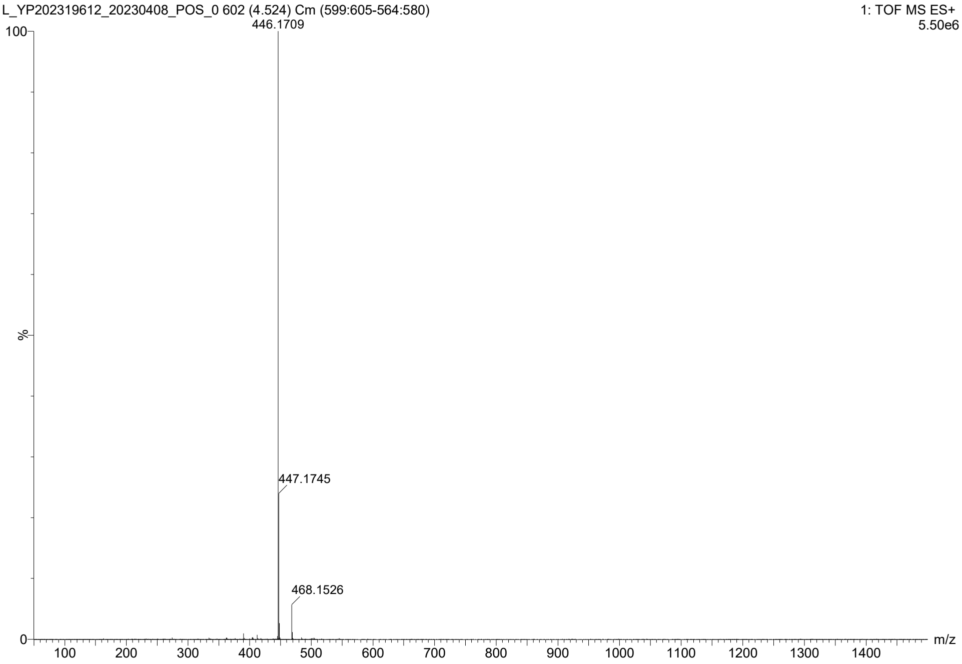

Figure S4. HRMS spectra of QC.

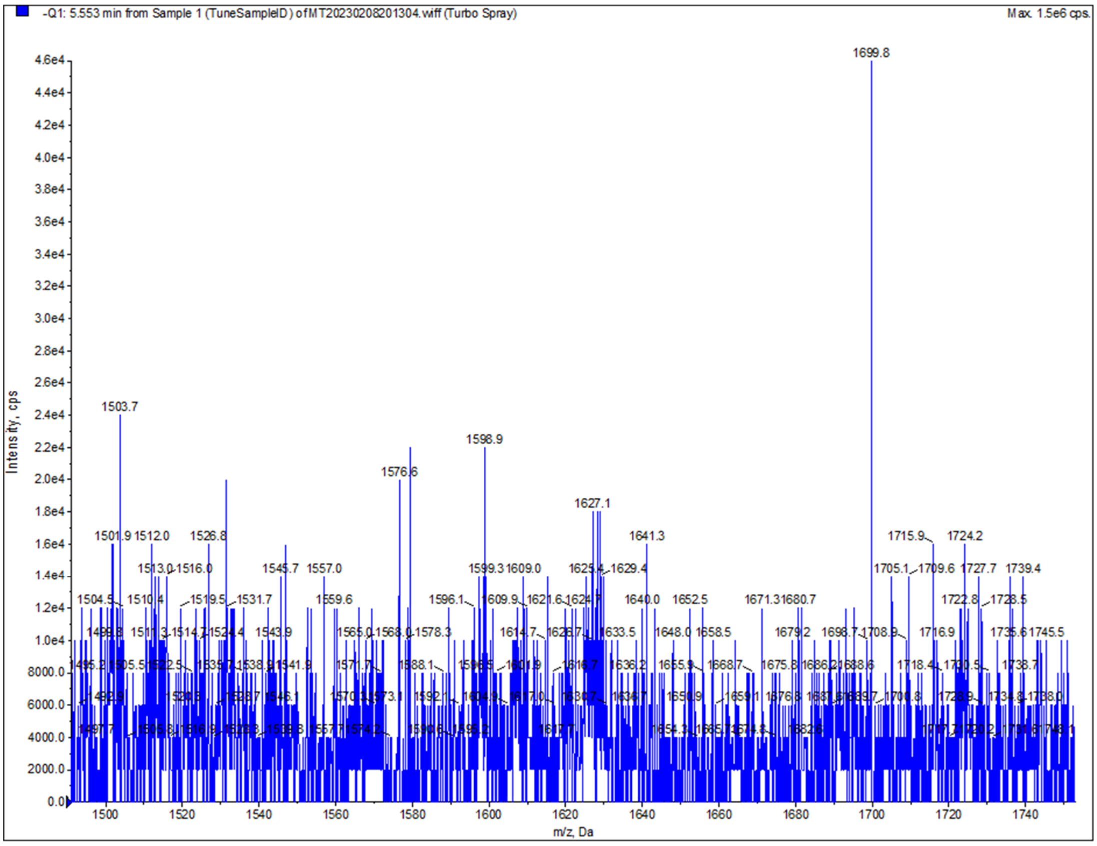

Figure S5. MS spectra of AC-QC nanoparticles.

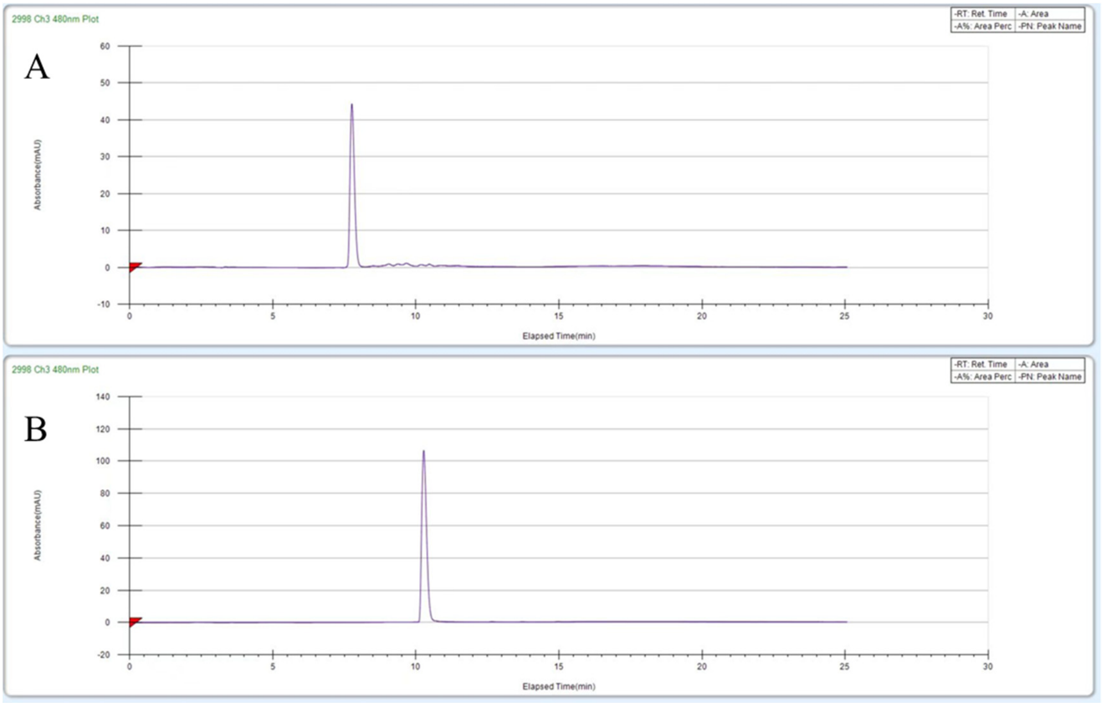

Figure S6. HPLC spectra of AC peptide (A) and AC-QC (B).

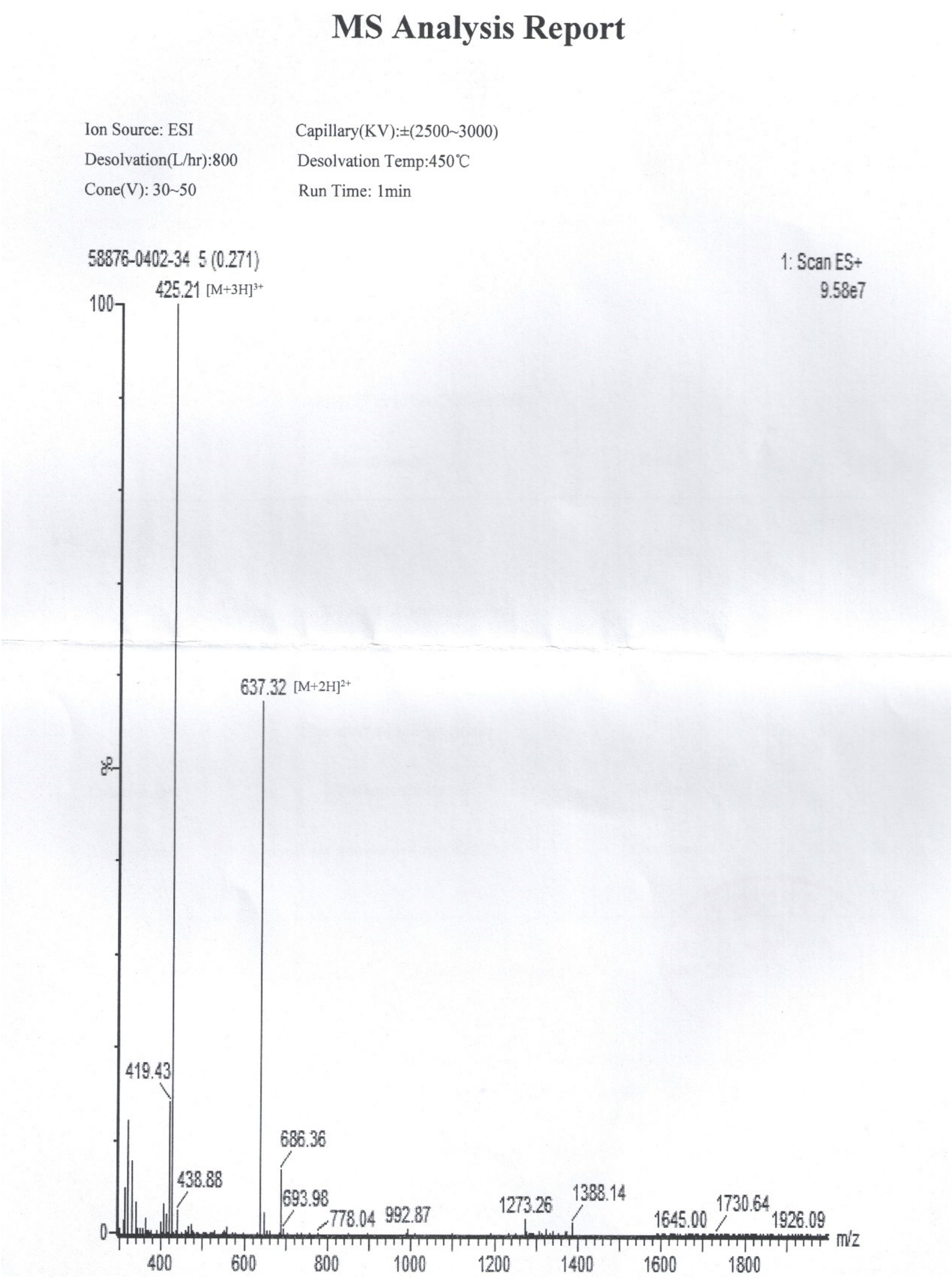

Figure S7. MS spectra of AC peptide.

Figure S8. QC fluorescence spectra in PBS solvents.

Figure S9. AC-QC fluorescence spectra in various solvents.

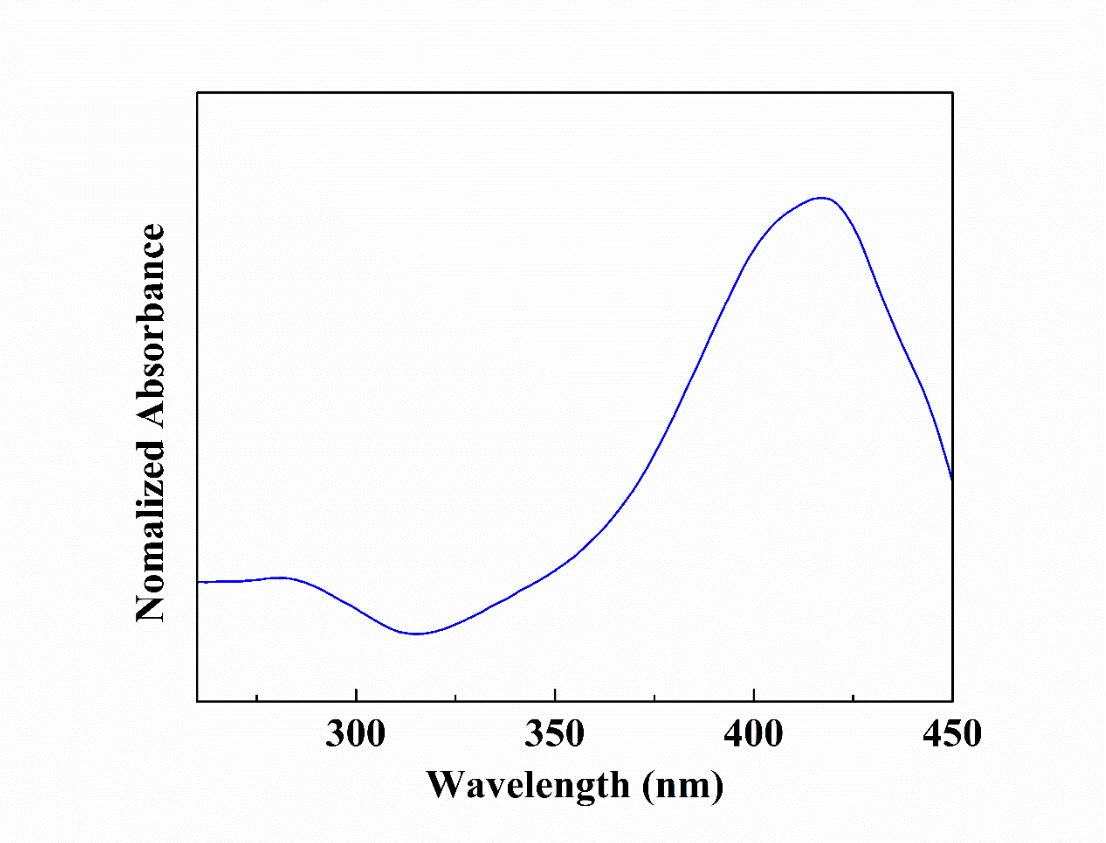

Figure S8 AC-QC conjugate of UV−vis absorption spectra in DMSO.

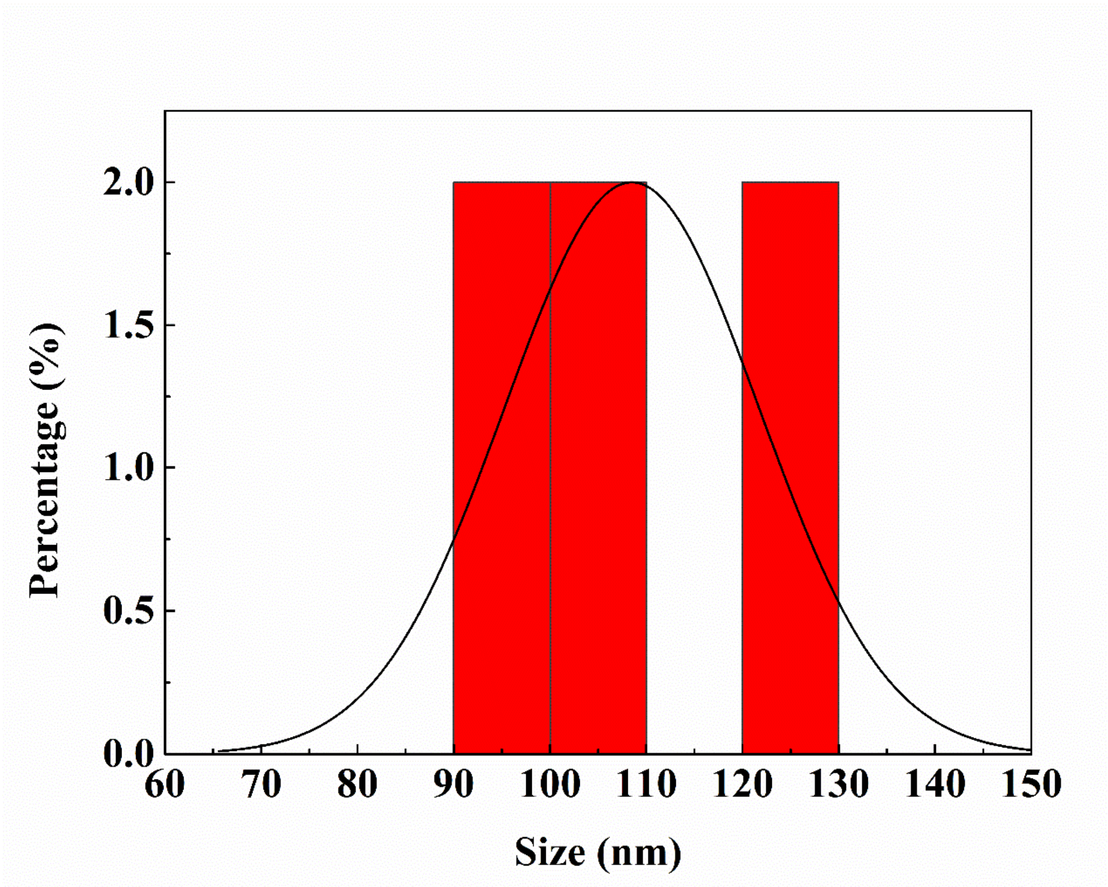

Figure S9. The size distribution of TEM.

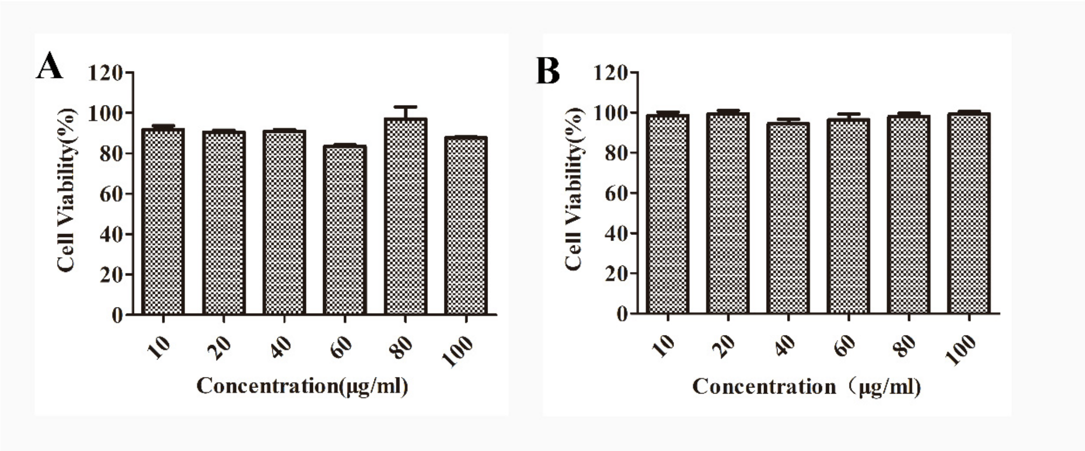

Figure S10. Relative cell viability of IOSE-80 cells (A) and L929 cells (B) against nanoparticle solution after cultured for 72 h with different nanoparticle concentrations.

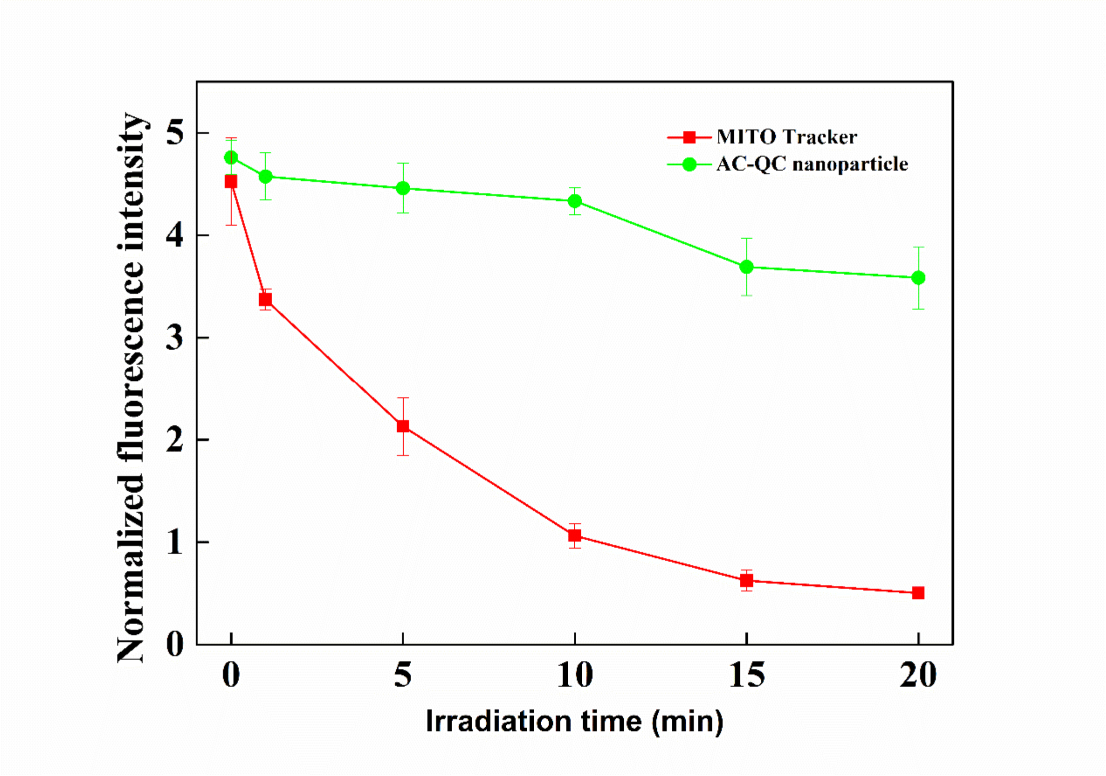

Figure S11. Time-dependent peak fluorescence intensity of MITO Tracker and AC-QC nanopatticle after 20 min irradiation. Data represent mean values ± standard deviation, n = 3, the statistical significance level is ***p < 0.001. All experiments were carried out three times independently.

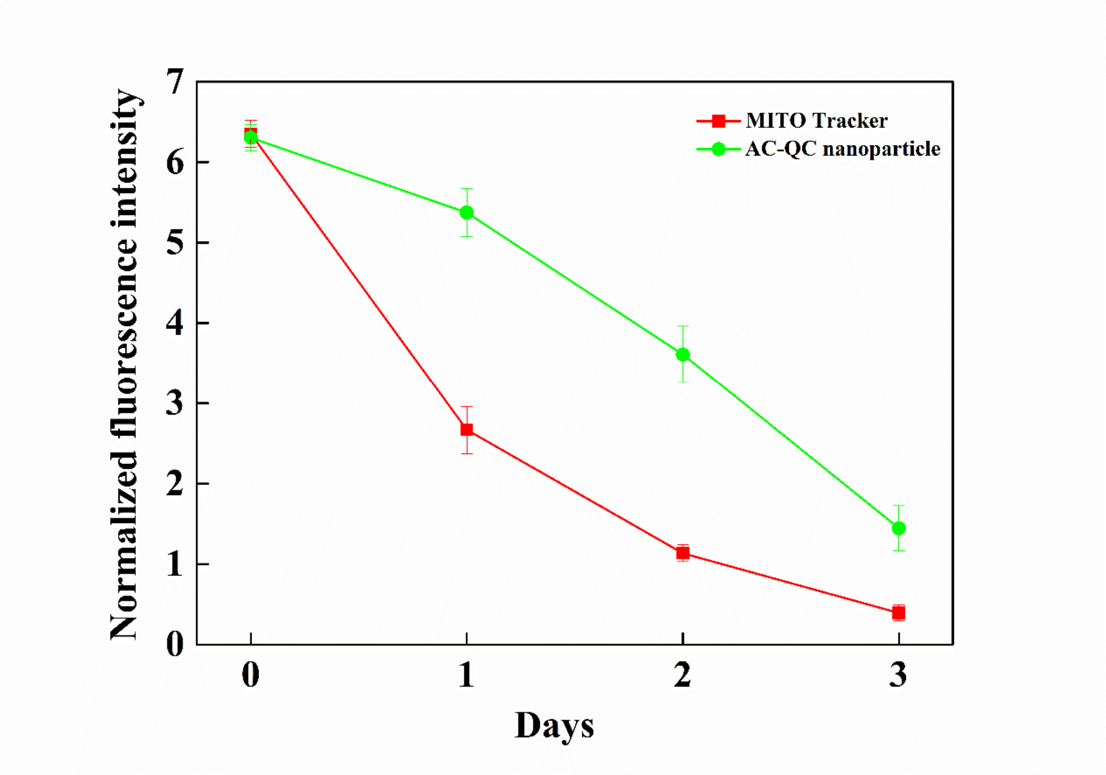

Figure S12. Long-term peak fluorescence intensity of MITO Tracker and AC-QC nanopatticle at 37 °C for 4 h and subculture for various time intervals from day 0 to day 3. Data represent mean values ± standard deviation, n = 3, the statistical significance level is ***p < 0.001.The experiment was carried out three times independently.
